# The Efficacy of Gum Arabic in Managing Diseases: A Systematic Review of Evidence-Based Clinical Trials

**DOI:** 10.3390/biom13010138

**Published:** 2023-01-09

**Authors:** Yamamh Al-Jubori, Nazik Tayfour Babiker Ahmed, Rawan Albusaidi, James Madden, Srijit Das, Srinivasa Rao Sirasanagandla

**Affiliations:** 1 College of Medicine and Health Sciences, Sultan Qaboos University, Muscat 123, Oman; 2 GKT School of Medicine, King’s College London, Great Maze Pond, London SE1 1UL, UK; 3 Department of Human and Clinical Anatomy, College of Medicine and Health Sciences, Sultan Qaboos University, Muscat 123, Oman

**Keywords:** *Acacia senegal*, *Acacia seyal*, gum arabic, natural product, systematic review, treatment

## Abstract

Gum arabic (GA) is a natural product commonly used as a household remedy for treating various diseases in the Sub-Saharan Africa region. Despite its claimed benefits, there has been a lack of research on the findings of current clinical trials (CTs) that investigated its efficacy in the treatment of various medical diseases. The aim of this systematic review was to study CTs which focused on GA and its possible use in the management of various medical diseases. A search of the extant literature was performed in the PubMed, Scopus, and Cochrane databases to retrieve CTs focusing on evidence-based clinical indications. The databases were searched using the keywords (“Gum Arabic” OR “*Acacia senegal*” OR “*Acacia seyal*” OR “Gum Acacia” OR “Acacia Arabica”) AND (“Clinical Trial” OR “Randomized Controlled Trial” OR “Randomized Clinical Trial”). While performing the systematic review, data were obtained on the following parameters: title, authors, date of publication, study design, study aim, sample size, type of intervention used, targeted medical diseases, and main findings. Twenty-nine papers were included in this systematic review. The results showed that ingestion of GA altered lipid profiles, renal profiles, plaque, gingival scores, biochemical parameters, blood pressure, inflammatory markers, and adiposity. GA exhibited anti-inflammatory, prebiotic, and antibacterial properties. GA has been successfully used to treat sickle cell anemia, rheumatoid arthritis, metabolic disorders, periodontitis, gastrointestinal conditions, and kidney diseases. Herein, we discuss GA with respect to the underlying mechanisms involved in each medical disease, thereby justifying GA’s future role as a therapeutic agent.

## 1. Introduction

**Figure 1:**
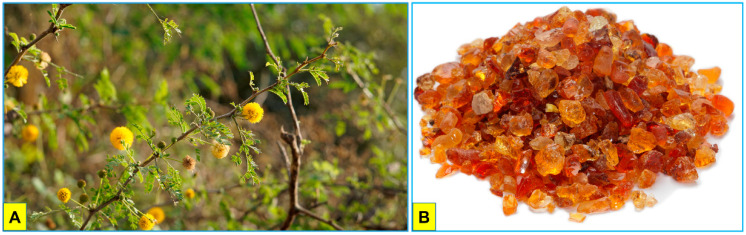
Photographs showing (**A**) *Acacia seyal* and (**B**) Gum Arabic.

**Figure 2:**
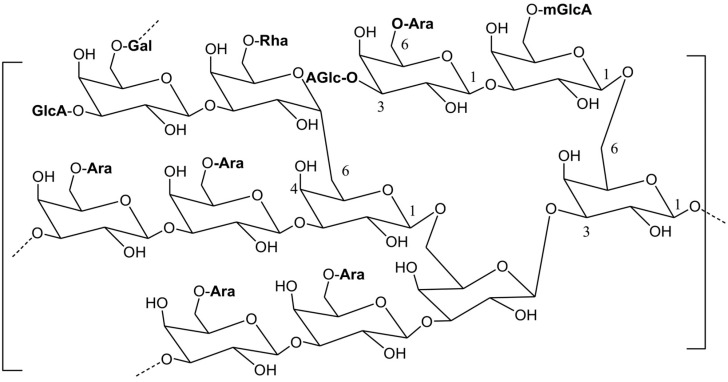
The basic chemical structure of arabic acid present in gum arabic [1].

Gum arabic (GA) is an exudate with a gummy texture obtained from *Acacia seyal* and *Acacia senegal* umbrella-shaped branches. A cut is made on the branches by which the exudate is obtained from or naturally present in and is made to harden in the air. GA is mainly found in Sudan, Chad, and Nigeria [1,2] (Figure 1). Structurally, GA is an arabinogalactan–protein complex. This complex is composed of magnesium, calcium, and potassium salts of arabic acid. Arabic acid structure is made up of 1-3-linked β-D-galactopyranosyl units, along with branches that consist of two to five β-D-galactopyranosyl residues linked together through 1,3-ether linkages and connected to the fundamental β-D-galactopyranosyl chain by 1,6-linkages (Figure 2) [1]. 

GA is largely fermented in the large intestines into short-chain fatty acids by microorganisms [3]. Traditionally, GA has been used as an oral hygiene substance [4]. Health benefits were seen following GA treatment. Direct application of herbal formulation containing GA on teeth and gums significantly reduced gingival and plaque index scores [4]. GA contains high amounts of calcium and phosphate ions. In vitro studies demonstrated that it can prevent tooth enamel demineralization, in addition to enhancing its remineralization [5,6,7]. In mice, GA supplementation in drinking water or along with diet was observed to reduce obesity by altering the expression of lipid metabolic genes and age-dependent fat deposition in the visceral adipose tissue [8,9]. It lowers cholesterol levels, as it possesses a high amount of fiber. GA treatment, along with atorvastatin, reduced total cholesterol, LDL, and triglyceride levels in patients with hyperlipidemia [10]. These effects subsequently decrease the risk of heart disease [10].

GA also exhibited antioxidant properties by increasing superoxide dismutase, catalase, and glutathione peroxidase activity in the liver [3,11,12,13]. GA oral supplementation increased 24 h creatinine clearance and binds with free water, thereby reducing intestinal absorption and water content in urine [14,15]. Surprisingly, GA was ignored by the locals, and the tree branches were used for coal and fire with decreases in production [1]. Thus, the benefits were unknown until recently, when many experimental and CT studies revealed its benefits.

Several experimental studies have demonstrated the potential benefits of the use of GA in clinical practice [16,17,18,19]. In a recent study, GA treatment inhibited colorectal carcinogenesis in mice [16]. GA treatment reduced the formation of aberrant crypts foci in the colon, mainly by reducing local genotoxicity, as well as oxidative stress [16]. In the same study, reduced genotoxicity in the liver and bone marrow, as well as low oxidative stress in the liver and blood, were observed [16]. GA supplementation protected rat heart from ischemia/reperfusion injury by decreasing apoptotic enzyme levels, as well as from the formation of proinflammatory cytokines [20]. GA treatment alleviated B_1_-induced hepatic injury through its antioxidant and anti-inflammatory properties [17]. 

Another research study showed that regular GA consumption stimulates innate immunity against various infections by inducing cathelicidin expression [18]. GA supplementation in type 2 diabetic rats prevented learning and memory loss. These effects were associated with increased expression of PGC-1a and ATP synthase β-subunit protein in the hippocampus [21]. GA administration in rats with dextran sodium sulfate-induced colitis resulted in a reduction in the severity of colitis, colonic fibrosis, and TGFβ1 expression [22]. GA pretreatment prevented butralin-exposure-induced renal damage by promoting antioxidants and increasing free radical scavenging activity [23]. Another experimental study on diabetic rats concluded that GA reduced the progression of chronic kidney disease [24].

Studies were published on GA and water pipe smoking (WPS) in mice. Researchers showed that in male mice exposed to hookah smoke for 30 min each day for 30 days with coadministration of GA, the negative effects of smoke exposure on the reproductive system were reduced [25]. Another study on mice exposed to WPS and GA concluded that GA reduced the harmful effects of WPS on thrombosis, cardiovascular toxicity, inflammation, and oxidative stress [26].

GA was found to be effective against diarrhea [27,28]. GA supplementation as an additive to oral rehydration solution significantly reduced the duration of diarrhea and frequency of defecation and improved the consistency of the stool [27]. Clinical trials (CTs) are being conducted in the human population to develop a new therapeutic drug in order to treat, prevent, or reduce the incidence of disease [29]. To the best of our knowledge, the present systematic review may be the first of its kind to discuss CTs conducted on human subjects exclusively focusing on GA and its beneficial effects in the management of various medical diseases.

## 2. Materials and Methods

### 2.1. Study Design 

A systematic review of all human CTs was conducted to explore the current best evidence of the possible use of GA for various medical diseases.

### 2.2. Search Strategy 

Relevant studies were identified through a thorough search of electronic databases such as PubMed, Scopus, and the Cochrane library. In addition, a snowballing method was employed whereby relevant articles were found by screening the reference list for any additional articles that met the eligibility criteria of the current study. The earlier published papers were screened during the period from April 2022 to August 2022. The databases were searched using the keywords: (“Gum Arabic” OR “*Acacia senegal*” OR “*Acacia seyal*” OR “Gum Acacia” OR “Acacia Arabica “) AND (“Clinical Trial” OR “Randomized Controlled Trial” OR “Randomized Clinical Trial”). Unpublished articles were excluded from the search strategy.

### 2.3. Inclusion Criteria 

Published literature that fulfilled the following criteria was included: (i) all studies that were published in English language and reported CTs of GA treatment against targeted medical conditions in humans. All CTs of GA treatment, regardless of randomization, blinding, phase of trial, and statistical method, were used for assessment of outcome, irrespective of negative or positive results. 

### 2.4. Exclusion Criteria 

The exclusion criteria for the present systematic review were: “Studies that used other natural product/compound combined with GA for the treatment”; “any preclinical, unpublished, duplicated, and incomplete CTs”; “Studies that reported the GA CT without a specific targeted medical disease” and “Studies that were published in languages other than English”. 

### 2.5. Data Collection

Reviewers (Y.A.-J., N.T.B.A., R.A., J.M., and S.R.S.) first screened titles and abstracts of all retrieved papers for inclusion. The full texts of all screened papers were then studied independently in order to determine the final study selection. Duplicate information on the same studies was removed. Agreement on the inclusion and exclusion criteria was concordant, and discrepancies were resolved by consensus of all researchers. The following data were collected from included studies: authors and date of study, study design, targeted medical disease, sample size, and main results/findings (Table 1).

## 3. Results

### 3.1. Study Selection

This review was performed in accordance to the PRISMA (Preferred Reporting Items for Systematic Reviews and Meta-Analyses) guidelines.

**Figure 3:**
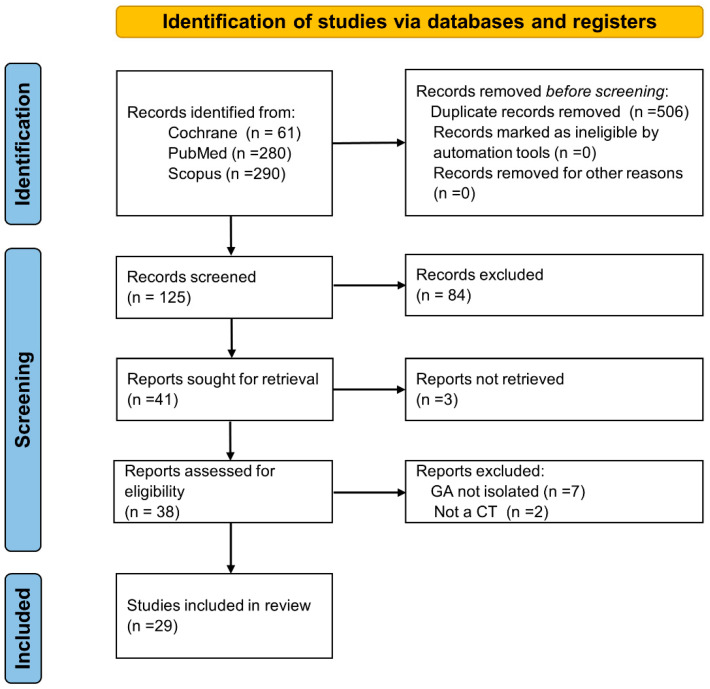
PRISMA flow diagram showing the selection, inclusion, and exclusion of clinical trial studies.

The literature search led to the identification of 631 studies (Figure 3). Following an initial screening for duplicates and after applying the inclusion criteria to the title, the abstracts of 38 articles were found to be suitable for full-text screening. During title screening, the agreement between the six reviewers was unanimous and conclusive. Twenty-nine articles were eligible, as they met the inclusion criteria and were therefor included in the systematic review.

### 3.2. Study Characteristics

The majority of the publications (79.3%) were published after 2010. The papers included information from nine different countries. The studies included in the systematic review were from worldwide populations in Asia, Africa, Europe, and the United States. All the studies included in this review were CTs. Of the 29 articles that were included, 31.0% were related to metabolic disorders (e.g., type 2 diabetes and hyperlipidemia) [10,30,31,32,33,34,35,36,37], 13.9% were related to kidney diseases [13,14,50,51], 17.2% were related to gastrointestinal health [49,52,53,54,55], 17.2% were related to oral health [41,42,43,44,45], 10.3% were related to sickle cell anemia [38,39,40], 6.9% were related to rheumatoid arthritis [46,47], and 3.4% were related to drug efficacy [48] (Table 1).

### 3.3. Gum Arabic and Other Diseases 

The findings from different published studies are discussed under different sections.

#### 3.3.1. Gum Arabic and Metabolic Disorders 

GA was found to have a positive effect on satiety and appetite reduction [30]. Subjects reported a decrease in caloric intake and an increase in the their satiety following consumption of GA [33]. Furthermore, when studying the effects of ingesting GA on adults who were at high risk of developing metabolic syndrome, it was found that study subjects had reduced systolic and diastolic blood pressure, fat-free body mass, appetite, and fasting plasma glucose, along with an increased dietary fiber intake. Improvement in bloating and bowel movements was also reported [34].

Another study asserted similar findings; subjects who ingested GA exhibited a significant reduction in fasting plasma glucose and HbA1c [34,35,36]. Another trial, in which patients with hyperlipidemia were given GA alongside atorvastatin medications, reported that the reduction in their lipid profile was significantly improved [10]. A significant increase in the HDL cholesterol level was also noted [35]. 

These findings were contradicted by one study that investigated the effects of the viscosity of fiber supplements on the lipid profile. The authors discovered that GA, owing to its low viscosity, did not significantly lower cholesterol levels compared to other fiber supplements containing medium- to high-viscosity water-soluble dietary fiber [37]. Moreover, GA was deemed useful for the reduction in weight gain in patients with type 2 diabetes, as it helped to decrease the body adiposity index by 23.7% and BMI by 2% [31]. Another study yielded similar findings; a reduction of 2.18% of body fat was reported in patients who ingested GA [32]. It was further observed that consumption of GA significantly decreases fasting blood glucose, HbA1c, total protein, and uric acid concentration [36]. It was also observed that GA is a helpful supplement for diabetic patients, specifically those who have diabetic nephropathy, as it decreases blood urea nitrogen and creatinine concentrations [36].

#### 3.3.2. Gum Arabic and Sickle Cell Anemia 

In sickle cell anemia, dyslipidemia is a common occurrence as a result of oxidative stress reactions [38]. It was revealed that GA significantly reduced total cholesterol, LDL, and triglyceride levels [38]. A team of researchers who conducted a CT study found that sickle cell patients who consumed GA experienced a significant reduction in their direct bilirubin, serum alanine transaminase, and serum urea levels [39]. Another beneficial effect of GA was an increase in total antioxidant capacity and a reduction in MDA and H_2_O_2_ oxidative markers in sickle cell anemia patients [40]. 

#### 3.3.3. Gum Arabic and Oral Health 

CTs were conducted to test the efficacy of GA as an antibacterial in comparison to liquorice and chlorhexidine mouthwashes. Results revealed a statistically significant decrease in the counts of *Streptococcus mutans* and *Lactobacillus acidophilus* for both GA and liquorice mouthwash without any oral side effects. Moreover, resistance was observed in subjects who used chlorhexidine mouthwash. No significant difference was found between GA and liquorice mouthwash, implying that they can both be used to effectively prevent dental caries [41].

Furthermore, another research finding in the field of oral health showed the effective use of GA in the prevention of dental plaques [42]. In comparison to sugar-free gum, daily photographic assessment of erythrosine-stained plaque showed lower scores following consumption of GA [42]. A group of researchers observed similar findings with respect to reduction in plaque and gingival inflammation [43]. Subjects who applied GA powder had a significantly lower mean gingival index score, mean plaque index score, and gingival crevicular fluid interleukin-1β [43].

A CT study reported that GA use has been of immense benefit for patients with xerostomia. Subjects who were in the GA group had a significantly higher salivary flow rates by 8.03 g within 10 min compared to the control group [45]. Another CT study observed a significant reduction in PPD and a gain in CAL in subjects who used GA gel. Additionally, improved plaque and gingival index scores were also noted [44].

#### 3.3.4. Gum Arabic and Rheumatoid Arthritis 

It was found that GA had a positive effect on restoring the baseline liver and kidney profiles in patients with rheumatoid arthritis [46]. GA significantly decreased liver enzymes, with the exception of alkaline phosphatase, urea, and sodium levels, and significantly increased albumin levels, with a minor impact on the serum globulin level [46]. Another research study showed that GA significantly decreased TNF-alpha, the erythrocyte sedimentation rate, and the number of swollen and tender joints, as well as the disease severity in rheumatoid arthritis patients [47].

#### 3.3.5. Gum Arabic and Drug Interactions 

GA was also found to enhance drug efficacy, specifically the absorption of amoxicillin [48]. When measuring the peak amoxicillin concentration between two groups (one that took GA 2 h post amoxicillin ingestion and the other that took the drug simultaneously with GA), it was found to be significantly lower in the group that took GA simultaneously [48]. In an experimental study, the effect of GA on gastric ulcers and its interaction with the antiulcer effect of ranitidine was studied in rats. GA significantly potentiated the antiulcer effect of ranitidine [56]. In another study, oral administration of GA was shown to accelerate the absorption of certain solutes [57]. Meloxicam was used as an anti-Cox-1 and Cox-2 non-steroidal anti-inflammatory drug [58]. In rats, GA supplementation showed protective effects against meloxicam-induced gastrointestinal insult. In this study, there were no pharmacological interactions with meloxicam [58].

#### 3.3.6. Gum Arabic and Gastrointestinal Conditions 

Researchers observed that administration of GA positively improved acute non-bloody diarrhea in children in terms of symptoms, weight improvements, and the prevention of marked severe dehydration [54]. Treatment with GA was found to be ineffective in patients with fecal incontinence. Patients who received GA supplementation had a fecal incontinence frequency that was not statistically different from the group that received a placebo. Instead, psyllium as a supplement was found to be of beneficial in reducing the frequency of fecal incontinence [52]. Another CT study revealed a beneficial effect of GA in children who had colostomies. It was revealed that the group with GA ointment experienced a significant reduction in peristomal skin inflammation in comparison to the control group [49]. GA was also shown to have potential prebiotic benefits. Researchers found that following ingestion of GA, the count of Bifidobacterium and Lactobacilli increased significantly [53]. In a CT, patients with gastroparesis were shown to benefit from GA administration, as it played a role in the regulation of their blood glucose levels. However, no significant findings were noted with respect to the mouth-to-cecum transit delay [55].

#### 3.3.7. Gum Arabic and Chronic Kidney Diseases

**Figure 4:**
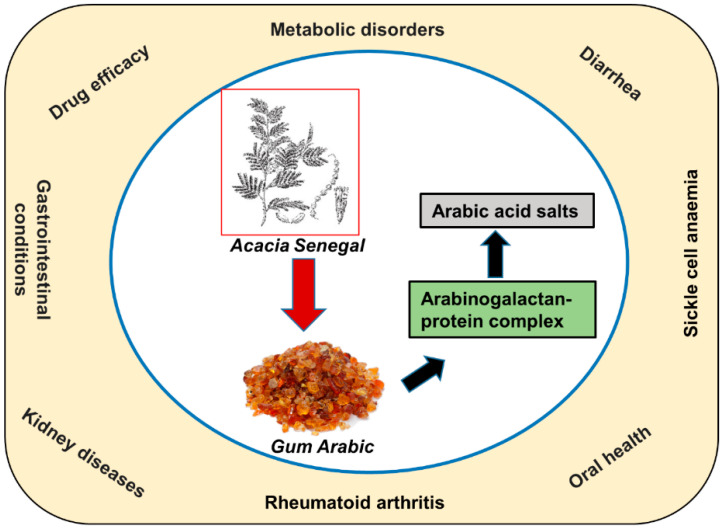
A schematic diagram depicting the beneficial effects of GA on various medical diseases.

GA was also found to alleviate the adverse effects of chronic renal failure [13,14,50,51]. Patients with chronic renal failure who received GA showed significant decreases in serum urea levels compared to the baseline and the control group [51]. Serum creatinine levels also significantly decreased in the groups of gum users compared to the control group. There was a significant decrease in the serum uric acid level compared to baseline. Serum calcium levels increased, and this increase was significantly different from the baseline and control groups. Serum phosphorus levels decreased significantly compared to baseline [51]. A reduction in serum urea level was also reported in another CT, alongside an increase in fecal bacterial mass and nitrogen content in patients following GA consumption [14]. A study revealed that GA significantly increased total antioxidant capacity levels and reduced oxidative markers MDA and C-reactive protein in patients undergoing hemodialysis, serving as evidence of potent anti-inflammatory properties of GA [13]. These results are similar to those reported in another study showing that consumption of GA significantly decreased C-reactive protein and sodium levels without affecting the levels of other electrolytes, urine volume, or indoxyl sulfate [50]. A schematic diagram depicting the beneficial effects of GA on various medical diseases is shown in Figure 4.

## 4. Discussion

Based on the existing findings, we highlight the importance of GA in various diseases. GA supplementation was shown to be effective against metabolic disorders [10,30,31,32,33,34,35,36,37], sickle cell anemia [38,39,40], kidney disease [13,14,50,51], and rheumatoid arthritis [46,47], as well as for the management of oral health [41,42,43,44,45] and gastrointestinal conditions [49,52,53,54,55]. GA treatment did not show any potential drug interactions [48]. Additionally, research studies revealed positive effects on appetite and a subsequent significant effect on lowering patients’ caloric intake following GA consumption [30,33,36]. Other studies demonstrated similar effects for other gum products [59,60]. Hence, further research is warranted to explore the exact mechanism of action of the active components present in GA. Contradictory results related to GA’s effect on blood glucose levels have been reported [30,34,35]. Hence, addition CTs in a larger population need to be conducted in order to determine the exact role of GA in blood glucose levels. 

GA is a soluble fiber; dietary fiber increases fecal bulk [61] and reduces the transit time [62]. Dietary fiber is important for combating obesity, and increased intake of dietary fiber has been associated with a reduction in BMI [63]. Dietary fibers have effects on satiety and blood glucose levels. An previous study showed that GA exhibited significant positive effects on satiety measures 15, 30, and 240 min following consumption [30]. The type and viscosity of fiber can have an impact on blood glucose levels after consumption, and variation in the amount of fiber consumed cannot consistently predict the resulting reduction in postprandial glycemic response [30].

In in animals [9,64] and humans, GA treatment has been shown to be effective against body weight and adiposity. Researchers found that GA supplementation significantly reduced BMI, body fat percentage, hip circumference, lipid accumulation product, and visceral adiposity index (VAI) [31,32]. Furthermore, reduced VAI was associated with impaired glucose and lipid metabolism, insulin resistance, and hypertension [31].Thus, it was concluded that GA’s positive effects in combating obesity may be related to its positive effect on satiety [30].

Studies confirmed GA effects on chronic conditions, demonstrating their repeatability [31,33]. These effects include a reduction in weight gain, blood pressure, and BMI, which are all positive indicators, strongly suggesting the use of GA as a supplement [31,33]. In addition, it has been demonstrated that GA ingestion leads to a decrease in total cholesterol, LDL, and triglycerides in patients with sickle cell anemia [38]. This lipid-lowering effect is useful, as dyslipidemia is common in patients with sickle cell anemia resulting from oxidative stress [38].

It has been suggested that GA lowers lipid levels. The mechanism behind this phenomenon is that GA binds to bile acids and reduces their absorption from the terminal ileum [65]. The fermentation process in the large intestine then makes the bile acids insoluble, thus promoting their excretion in stool [65]. De novo production of bile acids by the liver requires serum cholesterol. Thus, prolonged ingestion of GA may lead to a reduction in the cholesterol level in plasma [65]. Whereas this information supports the former hypothesis, the findings from the published literature retrieved for this review do not necessarily confirm or deny this phenomenon. Further studies with sufficient clinical data need to be considered when prescribing as a supplement for the treatment of medical diseases.

A study was conducted on 47 patients carrying hemoglobin SS (HbS); GA was administered in a dose of 30 g/day for a period of 12 weeks [39]. In patients with sickle cell anemia, GA administration decreased bilirubin, AST, and serum urea [39]. The results show that GA increased the level of HbF and significantly decreased the level of HbS [40]. The positive effect of GA was explained by the fact that GA degradation resulted in short-chain fatty acids, which, in turn, stimulated HbF expression in the red blood cells [40].

It has not yet been confirmed whether GA directly causes these effects or whether they are byproducts of the repeatedly demonstrated effects on dietary content and overall caloric intake. However, this may be less likely, given that the effects lasted for eight weeks after patients discontinued treatment. Additionally, GA was found to significantly increase total antioxidant capacity and decrease MDA and H_2_O_2_ levels in patients with sickle cell anemia [40]. A similar effect was also found in patients undergoing dialysis, whereby treatment with GA led to decreased CRP and increased antioxidant capacity and MDA levels [51]. GA also resulted in decreased post-colostomy peristomal skin inflammation in pediatric patients treated with acacia ointment compared to those treated with zinc sulfate ointment [49]. Although this does not directly apply to the other studies because of the different mode of delivery, it does potentially provide insight into the mechanism of action of the active compounds in GA, which need to be studied in detail [49]. 

In addition to specific mechanisms, GA’s general anti-inflammatory [66] and antioxidant effects [11,13] are important in clinical conditions, especially following surgery. GA was also demonstrated to decrease serum urea, creatinine, uric acid, and phosphorus levels, and to increase serum calcium [51], in accordance with its positive effects on kidney product profiles reported in other studies involving various chronic kidney diseases [50]. 

It is pertinent to mention that DNA damage in kidney disease was first detected in the deoxycorticosterone acetate (DOCA)/salt model, and researchers found DNA single- and double-strand breaks [67]. Oxidative stress leads to damage of the kidneys [68]. The antioxidative properties of GA can verify the complete formation of superoxide and oxidative-stress-induced DNA double-strand breaks [11]. The potent antioxidant properties of GA can be used in vulnerable patient populations with various clinical conditions characterized by increased lipid peroxidation and tissue injuries [37]. However, potential drug interactions need to be better characterized, findings need to be explored in CTs with larger sample sizes.

GA was found to have a potential prebiotic effect, as researchers have demonstrated increased counts of Bifidobacteria and Lactobacilli in patients taking GA [53]. This can be explained by the fact that GA is only degraded in the cecum, where it undergoes complete fermentation and therefore promotes bacterial proliferation [53]. Fecal incontinence (FI) is loss of control of bowel contents, leading to discharge of fecal matter. Dietary fiber can lessen FI through its withstanding capacity to fermentation by colonic bacteria, as well as its solubility and degradation [52,69]. In a single-blind RCT, GA supplementation did not significantly reduce FI frequency compared to *psyllium* supplementation [52]. Researchers hypothesized that the high degradation of GA by colonic bacteria [69] and its subsequently reduced content in feces could be the reason for its lack of clinical effect on FI [52].

Inflammatory state affects the synovial joints [70]. In patients with rheumatoid arthritis, GA treatment improved liver and kidney enzyme profiles, with positive subsequent improvement in their condition [46]. Other findings reported decreased TNF-α and ESR; patients also experienced fewer swollen and tender joints and lower disease severity scores [47]. GA may act as a positive immunomodulator. Butyrate is an end product of dietary fiber and starch after their aerobic fermentation by colonic bacteria [71]. Butyrate is a well-known potent anti-inflammatory agent. It suppresses the expression of proinflammatory cytokines by inhibiting NF*κ*B activation [71].

The anti-inflammatory property of GA manifested through its derivative, butyrate. GA can be used as a natural means of increasing the level of short-chain fatty acids, which have an immunomodulatory effect that is helpful in reducing inflammation and improving patients’ quality of life [47].

GA was demonstrated to improve amoxicillin absorption [48]. Although no specific mechanism was suggested in the study, this result potentially indicates that GA affects the absorption of biomolecules in the gut, which may explain some of the effects observed in other studies [48]. This may amplify allergies to amoxicillin by increasing its serum concentrations with the same dose, which still needs to be considered if GA becomes more widely used. This effect was further substantiated by a study that found that the coexistence of GA and amoxicillin in the upper gastrointestinal tract significantly decreased the absorption of amoxicillin [48]. This might lead to therapeutic failure and the development of drug resistance [48].

Numerous research papers have found a possible relationship between GA and oral health [41,42,43,44,45]. A recent CT on GA mouthwash showed promising caries-preventive and antibacterial effects with no oral side effects. Furthermore, a lack of significant difference between oral *Streptococcus mutans* and *Lactobacillus acidophilus* counts, as well as DMF index scores, in patients treated with either a licorice mouthwashes or GA mouthwash indicated equivalent capabilities of the two interventions in preventing caries [41]. The same study demonstrated bacterial resistance and oral side effects to a chemical agent, chlorhexidine mouthwash, after 9 and 12 months of use. This demonstrates that GA may be used as a natural mouth wash to prevent caries and may have benefits associated with improved adherence. However, in our opinion, future studies are needed to ensure that other bacteria-causing caries are also prevented by GA. In addition, GA supplementation was found to be associated with increased enamel hardness, which may be explained by the presence of polysaccharides and the high concentration of minerals (calcium, magnesium, and sodium) in GA [5,6,7]. Other findings are associated with oral health, including a decrease in plaque formation compared to intervention with sugar-free gum [42,43,44]. This suggests that GA is also a suitable supplement that can be used to effectively prevent oral infections, in particular in patients with difficulty in brushing teeth (e.g., Parkinson’s patients living alone) or in areas where access to running water or dental hygiene products is limited [72].

The present study is subject to some limitations. We did not assess the reported CTs in terms of randomization, blinding, or phase of trial (0-V). Furthermore, the CTs were not scored in terms of quality. We did not limit the literature search to any particular time period but included as many studies as possible. Moreover, we did not assess the included published papers for publication bias. 

## 5. Recommendations

The majority of the reported CTs on GA are limited by the following principal factors: a lack of a specifically demonstrated mechanism of action; a lack of repeatable findings; and studies involving special populations, for example, pregnant and breast-feeding women, children, and elderly patients. Addressing these issues will provide robust evidence for the use of GA in therapeutic applications. GA has been reported to possess various compounds. The isolation of active compounds from GA is highly recommended because the efficacy of active compounds can be easily studied at the molecular or genetic level. Furthermore, this can ease the marketing of the drug as an effective candidate for therapeutic use. The reported CTs used different doses of GA. Further research is needed on the selection and preparation of the final dosage of GA specific to each disease. For effective results, additional focus should be placed on nanoformulation-based drug delivery of GA. An example is resveratrol nanoformulations, which have been reported to lead to remarkable results [73]. The CTs include in this review did not report any toxicities or side effects associated with GA treatment. The majority of included CTs are of short duration and therefore may not have revealed toxicities or side effects. Hence, future multicenter studies with longer durations of treatment and larger sample sizes are warranted.

## 6. Conclusions

This systematic review represents a humble attempt to justify the role of GA in complementary medicine with evidence from published CTs. The results of the included CTs reported on the efficacy of GA against various diseases, such as sickle cell anemia, rheumatoid arthritis, periodontitis, metabolic disorders, kidney disease, oral health problems, gastrointestinal conditions, and peristomal skin inflammation. These findings indicate that GA exerted quantifiable benefits in a number of inpatient and outpatient cohorts. Admittedly, CTs did not provide sufficient data on the adverse effects of GA. Future studies need to explore each active compound present in GA, which has various protective functions in different medical diseases. Easy availability, compliance, and cost-effectiveness could encourage the use of GA with evidence from larger studies from different parts of the world. Additionally, toxicity changes in the liver and kidney need to be explored in detail.

## Figures and Tables

**Table 1:** Table showing the study characteristics of clinical trials conducted to show the beneficial effects of GA in various medical diseases.

Sl No	Authors and Year	Treatment Duration	Dose and Mode of Treatment	Sample Size	Medical Disease	Clinical Tests	Main Findings
1	Larson et al., 2021 [30]	6 weeks	0, 20, and 40 g of GA in orange juice, along with a bagel and cream cheese, after a 12 h fast	48	Type 2 Diabetes	Computerized visual analog scales	After the individuals consumed a bagel and cream cheese, along with 40 g of GA, they reported feeling less hungry 15 min and 240 min later. In comparison to the control group, the post-acacia ingestion symptoms of bloating, gas, and GI rumbling were more severe. Although there was no significant difference in the area under the curve or changes in blood glucose response, blood glucose with 20 g of fiber at 30 min was considerably lower than the control.
2	Babiker et al., 2018 [31]	3 months	30 g per day of GA administered orally	91	Type 2 Diabetes	Anthropometric obesity markers, blood pressure, and lipid profile	The effects of GA reduced BMI by 2% and visceral body adiposity index by 23.7%.
3	Babiker et al., 2012 [32]	6 weeks	30 g per day of GA administered orally	120	Obesity	Weight and height using standardized scales, skin fold thickness using a Harpenden skin fold caliper, and fat percentage using the Jackson and Pollock 7 caliper method and Siri equation	Body fat percentage reduced by 2.18%.
4	Calame et al.,2011 [33]	3 h post consumption	STUDY 1: 5 and 10 g of EmulGold (EG) and 10 and 40 g of PreVitae (PV) dissolved in 250 mL of water; STUDY 2: “glass of water containing 0, 10, 20 or 40 g of EG or PV”	70	Obesity	Caloric intake and visual analog scale	Three hours after ingesting GA, participants showed substantial reductions in calorie consumption and subjective feelings of fullness.
5	Jarrar et al., 2021 [34]	12 weeks	20 g daily oral dose of GA	61	Metabolic Syndrome	Anthropometric measurements, diet and physical assessment via The Food Processor^®^ nutrition and fitness software, ESHA food-analysis program (version 10.4), the Kuwaiti Food Composition database, bowel movement and satiety questionnaire, fasting blood sample to measure lipid profile, HbA1c, and fasting blood glucose	Systolic and diastolic blood pressure, fat-free mass, hunger, and fasting plasma glucose were all lower in the participants. An increase in dietary fiber consumption was reported. Bowel motions and bloating were improved.
6	Babiker et al., 2017 [35]	3 months	30 g daily intake of GA administered orally	91	Type 2 Diabetes	BMI, lipid profile, fasting blood glucose, and HbA1c	BMI was significantly lower in the GA group by 2.1%, although there was no significant change in waist size. Regarding metabolic measures, the GA group presented with a substantial reduction in fasting plasma glucose of 6.24% and a reduction in glycosylated hemoglobin (HbA1c) of 8.8%. By reducing LDL cholesterol by 19.5%, total cholesterol by 8.28%, and triglycerides by 10.95% from baseline values, GA supplementation improved lipid profiles. Within the GA group, HDL cholesterol significantly increased by 19.89%.
7	Nasir et al., 2016 [36]	16 weeks	10 g daily intake of GA administered orally	40	Type 2 Diabetes	Anthropometric measurements, lipid profile, fasting blood glucose, HbA1c, urea nitrogen, and creatinine	Fasting blood sugar and HbA1c levels significantly decreased in all groups, which was followed by large drops in total protein and uric acid levels. In diabetics and those with diabetic nephropathy, there was a noticeable improvement in renal function following GA supplementation across all groups, with substantial reductions in blood urea nitrogen and creatinine levels.
8	Haskell et al., 1992 [37]	12 weeks	15 g of GA dissolved in water	62	Hyperlipidemia	Lipid profile	Water-soluble dietary fiber made up of psyllium husk, pectin, guar and locust bean gums was significantly reduced in cholesterol—comparable to changes observed with 10 g of water-soluble dietary fiber per day from high-viscosity guar gum. GA (low viscosity) did not produce a significant lipid-lowering effect compared to a placebo.
9	Mohamed et al., 2015 [10]	4 weeks	30 mg of GA taken orally	110	Hyperlipidemia	Lipid profile	Reduced levels of total cholesterol (25.9% to 7.8%), triglycerides (38.2% to 2.9%), and LDL (30.8% to 8.1%) were the significant findings.
10	Kaddam et al., 2019 [38]	12 weeks	30 g per day of GA administered orally	47	Sickle Cell Anemia	Lipid profile	GA significantly decreased levels of triglycerides (10%), LDL (8% reduction), and total cholesterol (7% reduction).
11	Kaddam et al., 2019 [39]	12 weeks	30 g per day of GA administered orally	47	Sickle Cell Anemia	Liver enzymes, total protein, albumin, electrolytes, urea, creatinine, and uric acid were determined in the serum	Direct bilirubin, serum alanine transaminase, and serum urea levels all decreased significantly after 4 weeks of treatment, and these results persisted through the 8th week.
12	Kaddam et al., 2017 [40]	12 weeks	30 g per day of GA administered orally	47	Sickle Cell Anemia	Total antioxidant capacity (TAC), malondialdehyde (MDA), and hydrogen peroxide (H2O2) levels were measured by spectrophotometric methods	Total antioxidant capacity was significantly increased by GA, and oxidative indicators of MDA and hydrogen peroxide were reduced.
13	Kamal et al., 2020 [41]	1 year	62.5 mg of GA dissolved in 1 mL distilled water	63	Dental Caries	Mitis Salivarius agar and Lactobacillus MRS agar were used, and microbial analysis was performed	Chlorhexidine, GA, and liquorice mouthwashes differed statistically significantly from each other in the decayed, missing, and filled index (DMF). However, there was no statistically significant difference between the two latter groups. Patients who took GA or liquorice mouthwash had considerably lower levels of Streptococcus mutans and Lactobacillus acidophilus. However, there have been reports of oral adverse effects with chlorhexidine mouthwash.
14	Gazi, 1991 [42]	1 week	Chewing on GA for 10 min 5× a day	20	Plaque Formation	Gingival and plaque scores and daily photographic assessment of erythrocine-stained plaques	After GA compared to sugar-free gum, daily photographic examination of erythrocine-stained plaque revealed lower scores, as well as lower gingival and plaque scores.
15	Gafar et al., 2022 [43]	2 months	150 mg GA powder applied twice a day	30	Plaque-Induced Gingivitis	Plaque index, gingival index, and gingival crevicular fluid interleukin 1 beta were measured	It was discovered that GA worked well to reduce plaque and gingival irritation. The intervention group had considerably lower mean gingival index scores at 30 days, significantly lower mean plaque index scores at 30 and 60 days, and significantly lower gingival crevicular fluid interleukin 1 beta levels at 30 and 60 days.
16	Singhal et al., 2018 [44]	90 days	Participants were asked to use GA on a set of 2–4 teeth (premolars and molars) in one specified quadrant	59	Chronic Periodontitis	Periodontal pocket depth, clinical attachment levels, and gingival indices (GI) and plaque indices (PI)	In the group that utilized GA gel, there was a greater reduction in periodontal pocket depth and an increase in clinical attachment level, as well as a lower index of plaque and gingival index.
17	Bielfeldt et al., 2021 [45]	10 min	10 g of pastille GA	26	Xerostomia	Gravimetric measurement of salivary flow rate and visual analog scale. Saliva surface tension was measured in pooled saliva samples (0–5 min of sampling); Raman spectroscopy	After chewing the pastille, the mean salivary flow rate considerably rose by 8.03 g/min compared to the mean changes after chewing the control product, which increased by 3.71 g/min.
18	Kamal et al., 2021 [46]	12 weeks	30 g per day of GA administered orally	40	Rheumatoid Arthritis	A Cobas C311 (Roche, Germany) automated chemistry analyzer was used to directly determine the values for total protein, albumin, alanine aminotransferase (ALT), aspartate aminotransferase (AST), alkaline phosphatase (ALP), and creatinine	The liver enzymes ALT and AST were dramatically reduced due to GA, whereas ALP did not change appreciably. The albumin level significantly increased, whereas the serum globulin level minimally changed. GA considerably lowered urea and salt levels, with little impact on creatinine and potassium levels in terms of electrolyte levels and renal function.
19	Kamal et al., 2018 [47]	12 weeks	30 g per day of GA administered orally	40	Rheumatoid Arthritis	Complete blood count and liver and kidney function test	The levels of tumor necrosis factor alpha, erythrocyte sedimentation, the number of swollen and painful joints, and the illness severity score (28 P.V:0.00) were all shown to be significantly reduced by GA. Blood index changes were minimal.
20	Eltayeb et al., 2004 [48]	One-time oral dose	N/A	24	Drug Efficacy	Blood samples	When comparing two groups (one that took GA 2 hours after ingesting amoxicillin and the other that took the medication at the same time as GA), it was discovered that the latter group’s peak amoxicillin concentration was much lower.
21	Hosseinpour et al., 2012 [49]	4 weeks	GA ointment	60	Peristomal Skin Inflammation	Skin inflammation was diagnosed clinically according to a study by Ruzicka et al.; severity was defined according to the Physician Global Assessment (PGA)	Comparing the GA ointment group to the control group using zinc sulfate ointment, the GA ointment group showed a substantially lower level of peristomal skin inflammation.
22	Ali et al., 2020 [13]	12 weeks	30 g per day of GA administered orally	40	Kidney Disease	Blood samples	GA decreased the levels of the oxidative markers of MDA and CRP and significantly increased overall antioxidant capacity.
23	Elamin et al., 2017 [50]	4 weeks	10, 20, or 40 grams per day of GA administered orally	36	Kidney Disease	Clinical interviews for symptom severity, fasting blood sample, and 24 h urine collections	Both sodium levels and C-reactive protein levels were decreased in individuals who took GA. Other electrolyte concentrations, urine volume, and indoxyl sulfate levels were not affected.
24	Ali et al., 2008 [51]	3 months	50 g daily oral dose of GA	36	Kidney Disease	Blood samples	In comparison to baseline, GA was observed to significantly lower blood concentrations of urea, creatinine, uric acid, and phosphorus. GA ingestion was also reported to increase serum calcium levels.
25	Bliss et al., 1996 [14]	1 month	50 g of GA	20	Chronic Renal Failure	Blood and stool samples	The amount of nitrogen and bacterial bulk in feces dramatically increased due to GA. Additionally, serum urea nitrogen was significantly decreased.
26	Bliss et al., 2014 [52]	32 days	16 g daily intake of GA administered orally	189	Fecal Incontinence	Fecal collection and fecal incontinence diary filled in by subjects	The frequency of feces incontinence was comparable between individuals who took GA and those who took a placebo. It has been claimed that psyllium supplements can lower the frequency of fecal incontinence.
27	Calame et al., 2008 [53]	4 weeks	Daily doses (5, 10, 20, and 40 g) of GA (EmulGold^®^) dissolved in water	54	Prebiotic	Fecal samples and volunteer survey	In comparison to the negative control group, the counts of Bifidobacteria and Lactobacilli were significantly increased after GA administration—10 g in particular.
28	Salah et al., 2012 [54]	Until resolution (range: 24 h–6 weeks; average: 5 days)	5–10 mg GA solution administered daily	180	Acute Non-Bloody Diarrhea	Electrolytes, body weight, and clinical observation	90% of the children who took GA had their diarrhea cease within 24 hours, and 80% had been released after one day. Within the first five days of their admission, each of them made progress and was released. None of them had shock or severe dehydration. Only three children experienced electrolyte imbalance. At the conclusion of the trial, the weight had increased by 47.8% and had dropped by just 5.5%. Only two (3.3%) of the 61 children who were still being monitored after 6 weeks had experienced diarrhea again.
29	Suresh et al., 2021 [55]	1 month	10 g of GA dissolved in 200 mL of water	10	Gastroparesis	Weight and height, glucose measurement, and ANMS GCSI-DD validated survey for symptom severity	In comparison to psyllium husk, blood sugar levels were controlled in patients receiving GA and partly hydrolyzed guar gum. For the test fibers, the mouth-to-cecum transit time was not significant.

## Data Availability

Not applicable.

## Abbreviations

ATP	Adenosine triphosphate
BMI	Body mass index
CTs	Clinical trials
CRP	C-reactive protein
DMF	Decay–missing–filled
ESR	Erythrocyte sedimental rate
GA	Gum Arabic
HbA1c	Hemoglobin A1C
HDL	High-density lipoprotein
LDL	Low-density lipoprotein
MDA	Malondialdehyde
NPs	Natural products
NF-κB	Nuclear factor-κB
PGC-1alpha	Peroxisome proliferator-activated receptor-gamma coactivator
TGFβ1	Transforming growth factor beta 1
TNF	Tumor necrosis factor
WPS	Water pipe smoking
